# PTH1R-CaSR Cross Talk: New Treatment Options for Breast Cancer Osteolytic Bone Metastases

**DOI:** 10.1155/2018/7120979

**Published:** 2018-07-29

**Authors:** Yanmei Yang, Bin Wang

**Affiliations:** Center for Translational Medicine, Department of Medicine, Sidney Kimmel Medical College, Thomas Jefferson University, Philadelphia, PA 19107, USA

## Abstract

Metastatic breast cancer (BrCa) is currently incurable despite great improvements in treatment of primary BrCa. The incidence of skeletal metastases in advanced BrCa occurs up to 70%. Recent findings have established that the distribution of BrCa metastases to the skeleton is not a random process but due to the favorable microenvironment for tumor invasion and growth. The complex interplay among BrCa cells, stromal/osteoblastic cells, and osteoclasts in the osseous microenvironment creates a bone-tumor vicious cycle (a feed-forward loop) that results in excessive bone destruction and progressive tumor growth. Both the type 1 PTH receptor (PTH1R) and extracellular calcium-sensing receptor (CaSR) participate in the vicious cycle and influence the skeletal metastatic niche. Thus, this review focuses on how the PTH1R and CaSR signaling pathways interact and contribute to the pathogenesis of BrCa bone metastases. The effects of intermittent PTH and allosteric modulators of CaSR for the use of bone-anabolic agents and prevention of BrCa bone metastases constitute a proof of principle for therapeutic consideration. Understanding the interplay between PTH1R and CaSR signaling in the development of BrCa bone metastases could lead to a novel therapeutic approach to control both osteolysis and tumor burden in the bone.

## 1. Introduction

Breast cancer (BrCa) is the most common cancer and the second leading cause of cancer-associated death in women [1]. Because of the progress made in early detection and surgical treatment of the primary tumor, mortality in BrCa patients is increasingly linked to the metastatic disease. The incidence of bone metastases in advanced BrCa occurs up to 70%, and only 20% of those patients survive five years from the time of diagnosis of bone metastasis [2]. Patients with BrCa bone metastases have severe bone pain, fractures, hypercalcemia, spinal cord compression, and muscle weakness [3], and these skeletal-related events significantly degrade the quality of life. Bone metastases can be treated locally with radiation therapy or surgical therapy. Systemic treatments include hormonal manipulations, cytotoxic chemotherapy, and/or bone-targeted therapy. However, there is little hope of a cure for BrCa skeletal metastases. Current management of metastatic bone complications is limited to the use of antiresorptive drugs such as bisphosphonates and receptor activator of nuclear factor-*κ*B ligand (RANKL) inhibitors, but the osteolytic bone disease often progresses, and over 50% of patients treated with these drugs will have a recurrence with skeletal-related events [4]. These drugs only inhibit bone resorption but do not restore bone formation and may cause side effects such as osteonecrosis of the jaw and decrease of renal function [5–7]. It has been established that the concerted actions of the type 1 PTH receptor (PTH1R) and extracellular calcium-sensing receptor (CaSR) maintain systemic extracellular ionized calcium (Ca^2+^) physical homeostasis and lactation, which was acknowledged by a number of excellent reviews [8–10]. In this article, we summarize the progress of interplay between the PTH1R and CaSR signaling in a vicious cycle of BrCa bone metastases, and interference of these interactions could offer new treatment options of BrCa bone metastases and their complications.

## 2. PTH1R and Its Ligands

PTH-related protein (PTHrP), also known as PTH-like hormone (PTHLH), was originally identified independently by several groups as a causal factor in the humoral hypercalcemia of malignancy [11–14]. The 13 amino-terminal amino acids are highly homologous to those of PTH, and both bind a common PTH1R. PTH is produced by the parathyroid gland and circulates as an endocrine regulator for the maintenance of calcium homeostasis (Table 1). In contrast to PTH, PTHrP is expressed in almost all normal fetal and adult tissues and acts through paracrine or autocrine mechanisms to regulate development and cell differentiation. In addition, PTHrP has the nuclear localization sequence and also acts via intracrine action to promote BrCa cell proliferation.

There are some conflicting reports on PTHrP function in primary tumors. While circulating levels of PTHrP positively correlate with the more advanced stages of cancer, some clinical studies indicated a good prognostic value for PTHrP in BrCa with less bone metastasis [15]. However, the *PTHrP* gene has recently been identified in a genomic locus associated with BrCa susceptibility [16]. Furthermore, Li and colleagues examined the role of PTHrP expression in animal models of BrCa and found PTHrP drove breast tumor initiation, progression, and metastasis in mice [17]. Taken together, PTHrP contributes to the pathogenesis of BrCa osteolytic bone metastases.

There are two types of the PTH receptor, PTH1R and PTH2R. The PTH1R and PTH2R belong to class B of the superfamily of G protein-coupled receptors (GPCRs) (Table 2). While PTH2R is mainly expressed in the central nervous system, PTH1R is present primarily in the kidney and bone [18] and is also located in the cartilage and breast. Like other GPCRs, the PTH1R activates multiple downstream signaling cascades by coupling to 4 major groups of G proteins, G*α*s, G*α*q, G*α*i, and G_12/13_. Interaction with cognate ligands of PTH1R, PTH and PTHrP, or biologically active peptide fragments, such as PTH(1–34), results in activation of G*α*s and G*α*q with consequent stimulation of adenylate cyclase and phospholipase C (PLC) [19, 20]. The action of PTH is also mediated through phospholipase D and mitogen-activated protein kinases. A cascade of cell-specific events of PTH mediates PTH1R function to regulate extracellular mineral ion homeostasis and bone remodeling.

## 3. CaSR and Its Ligands

The extracellular calcium-sensing receptor (CaSR) belongs to class C of GPCR that signals in response to Ca^2+^ and other ligands, such as gadolinium, polypeptides, and certain antibiotics [21, 22]. CaSR is expressed in the parathyroid glands, kidney, bone, normal breast epithelial cells, and BrCa cells [23]. Importantly, BrCa cells spread to the skeleton and express more CaSR than the cells in the primary tumor do [24]. Activation of the CaSR on BrCa cells contributes not only to BrCa cell proliferation and migration but also to the skeletal bone lesions.

In physiological condition, when the circulating Ca^2+^ level is low, the activation of CaSR in the chief cells of the parathyroid glands is reduced and PTH secretion is subsequently increased. PTH binds to the PTH1R and initiates a cascade of events that enhances renal tubular reabsorption of calcium, increases renal synthesis of 1,25(OH)_2_D_3_ so as to promote Ca^2+^ absorption in the intestine, and facilitates osteoclastic bone resorption, thereby maintaining Ca^2+^ homeostasis [9].

In normal mammary epithelial cells, activation of the CaSR during lactation inhibits PTHrP synthesis and secretion, thereby regulating maternal calcium and bone metabolism. In contrast, in BrCa cells, the Ca^2+^ released from bony matrix destruction binds to CaSR and stimulates PTHrP secretion [25]. This contradictory function is possible due to the ability of the CaSR to bind and activate different G protein subunits to switch from activation of the pertussis toxin-sensitive G*α*i and suppression of cAMP levels in the normal mammary epithelial cells to activation of G*α*s and stimulation of cAMP levels in BrCa cells in a cell type-specific manner [26].

## 4. Role of PTH1R and CaSR in the Bone-Tumor Vicious Cycle

**Figure 1:**
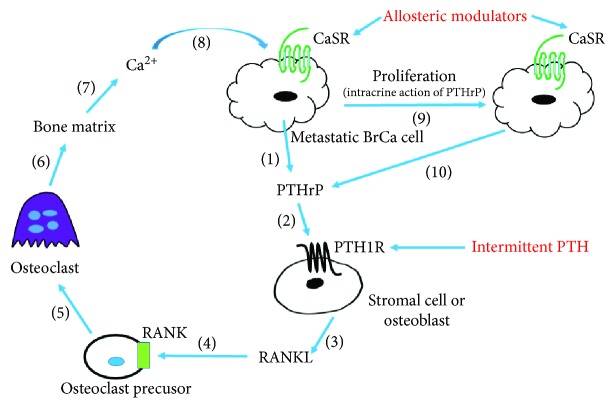
Interplay between PTH1R and CaSR plays critical roles in the pathogenesis of BrCa bone metastases. Numbers in parentheses indicate the event sequence during the formation of BrCa bone metastases. Treatment targets shown in red are likely to inhibit BrCa proliferation, increase osteoblast bone formation, and/or decrease osteoclast bone resorption.

Tumor metastasis to the secondary site is not a random event but is due to the favorable microenvironment [27, 28]. As early as 1889, Stephen Paget developed the “seed and soil” theory to describe the organ-specific metastasis, which indicates the distribution of tumor cells to certain organs largely depends on the specific feature of metastatic tumor cells (“seed”) and particular host microenvironment (“soil”) [29]. Primary breast tumors express CXCR4 [30], one of the metastasis markers, and secrete PTHrP. Li et al. reported that *PTHrP* ablation was accompanied by inhibition of CXCR4 expression in primary breast tumors, suggesting PTHrP is involved in the control of CXCR4 expression and consequently plays an important role in metastatic spread [17]. Osseous marrow stromal cells and osteoblasts secrete many chemokines including CXCL12 [31], which attracts CXCR4 positive BrCa cell homing and colonization to the bone. In response to the bone microenvironment, BrCa cells metastatic to the skeleton produce more PTHrP than the cells in the primary tumor [32]. Bone marrow stromal cells and osteoblasts, but not osteoclasts, express PTH1R. PTHrP binds to PTH1R mostly to induce G*α*s/cAMP signaling, which begets RANKL secretion (Figure 1). RANKL binds to its receptor RANK on osteoclast precursor cells and induces the differentiation and maturation of osteoclasts. The activated osteoclasts stimulate bone resorption and subsequent bony matrix destruction. Elevated extracellular Ca^2+^ released from the resorbed bone binds to CaSR on metastatic BrCa cells in the bone microenvironment (Figure 1). Unlike normal breast cells, CaSR activation induces G*α*s/cAMP pathway in BrCa cells and elicits further PTHrP production [26]. Furthermore, growth factors such as transforming growth factor *β* and insulin-like growth factor 1 that are stored during bone formation are released at sites of bone resorption and synergize with the effects of Ca^2+^ on CaSR to facilitate PTHrP secretion and worsen osteolysis [33, 34]. Because of its nuclear localization sequence, PTHrP can also act as an intracrine factor to promote tumor proliferation [21] that is independent of PTH1R (Figure 1) and then augment bone turnover, thereby driving the bone-tumor vicious cycle. Thus, the PTHrP-PTH1R interaction initiates the vicious cycle, and the subsequent Ca^2+^-CaSR signaling amplifies the manifestation of bone metastases, which in turn upregulates PTHrP production, thus setting up a feed-forward loop and exacerbating the osteolytic disease. Therefore, the interplay of PTH1R and CaSR acts in concert to evoke excessive bone destruction and progressive tumor growth.

## 5. Targeting the PTH1R and CaSR Signaling for Prevention of BrCa Bone Metastases

Generally, interference with each component or individual downstream signaling of the bone-tumor vicious cycle will have effects on the treatment of BrCa metastatic bone lesions. Bisphosphonates or RANKL inhibitors are antiresorptive drugs representing the current standard supportive treatment for BrCa bone metastatic complications. Due to a rapid action on inhibition of osteoclast activity, calcitonin may be used to lower the serum calcium levels before antiresorptive drugs exhibit their action in hypercalcemia occurring in BrCa patients. However, these drugs fail to enhance osteoblastic functions, which are impaired in BrCa patients. Increasing evidence has demonstrated that osteoblasts play a pivotal role in the pathogenesis of BrCa cell homing and colonization to the bone and subsequent metastatic bone lesions [35–37]. The ideal solution for treatment of BrCa bone metastases and their associated complications are (1) to block metastatic BrCa cell growth, (2) to generate an unfavorable bone microenvironment for BrCa cell colonization, and (3) to target the upstream signaling in the tumor-bone vicious cycle. Targeting PTHrP-PTH1R and Ca^2+^-CaSR signaling cascades meets these criteria and will generate new treatment options for prevention of BrCa metastases to the skeleton.

### 5.1. Intermittent Recombinant PTH(1–34) or Synthetic PTHrP(1–34) Analog

The bone is a metabolically active organ that undergoes continuous remodeling through the concerted actions of osteoblastic bone formation and osteoclastic bone resorption [38]. The bone remodeling balance is shifted toward bone destruction when metastatic BrCa cells invade and grow within the bone microenvironment. Both the disease of BrCa and current cancer treatment cause bone destruction during the BrCa progression [39]. Such bone loss occurs more rapidly to a greater degree than normal age-related osteoporosis [40]. Gregory et al. reported that the changes in bone formation and bone resorption activities were different at early and late stages during development of the bone lesion following intratibial injection of MDA-MB-231 human BrCa cells into the tibiae of athymic nude mice [36]. They found out that the early bone loss in the mouse models is due to a significant reduction in new bone formation by osteoblasts rather than increased levels of bone resorption by osteoclasts, indicating osteoblasts play a critical role in the early pathogenesis of BrCa bone metastasis. However, the current treatment of bone metastatic destruction is largely dependent on bisphosphonates or RANKL inhibitors, which only impede osteoclastic bone resorption but fail to increase osteoblastic bone formation. Thus, an alternative to antiresorptive drugs is anabolic therapy by targeting osteoblasts to promote bone formation.

Long after Bauer and colleagues discovered the anabolic effect of PTH in 1929 [41], recombinant parathyroid hormone (PTH)(1–34) (teriparatide, hereafter referred to as PTH) was approved as the first anabolic agent for the treatment of osteoporosis in the United States in 2002 [42]. PTH activates multiple signaling pathways, but not all of them are anabolic. Synthetic PTHrP(1–34) analog (abaloparatide) was approved in 2017 for osteoporosis therapy in an attempt to improve the anabolic effects of PTH1R signaling [43]. Both PTH and PTHrP exert either an anabolic or a catabolic effect depending on their doses and time duration of treatment [44, 45]. Intermittent administration of low-dose PTH or PTHrP increases bone formation, whereas continuous infusion of a high dose of PTH or PTHrP causes bone resorption and hypercalcemia [44, 46, 47]. While anabolic PTH effects on the bone are mediated through the cAMP/PKA signaling pathway [48, 49], PLC/PKC signaling has been shown to be inhibitory to the osteoanabolic actions of PTH [50]. It is also known that Wnt/*β*-catenin signaling [51] and other signaling pathways including phospholipase D [52, 53], ERK1/2 [54], and PI3K/AKT [55, 56] contribute to the anabolic PTH action in the bone.

Multiple myeloma (MM) is a hematologic malignancy of plasma cells, and osteolytic bone disease is the most common complication of MM [57]. Bone cells are directly involved in survival and expansion of myeloma cells in the hematopoietic bone marrow [58, 59]. Since signaling through the PTH1R in the osteoblast lineage regulates bone marrow hematopoietic niches, Pennisi et al. examined whether treating MM with an osteoblast-activating agent, intermittent PTH, could simultaneously help control bone disease and myeloma cell growth [60]. They demonstrated that PTH was capable of increasing bone mass in myelomatous bones *in vivo* and that the increased bone formation was associated with reduced tumor burden. The strategy that stimulation of osteoblast activity inhibits MM growth has received continuing interest for the treatment of solid tumors, such as BrCa and prostate cancer [61, 62]. Wu and colleagues demonstrated for the first time that intermittent PTH reduced the incidence of BrCa bone metastases in multiple mouse models. They found out that intermittent PTH decreased skeletal metastases and improved survival in the metastatic BrCa mouse model by injection of murine 4 T1 BrCa cells into the mammary fat pads. They further indicated that PTH administration retained its beneficial effect on tumor metastasis by increasing bone formation, decreasing osteoclast formation, and significantly reducing tumor engraftment and tumor burden of both murine and human BrCa cells in the mouse intratibial models. Since the CXCR4/CXCL12 axis has been established to play an important role in the homing of cancer cells to the bone [30, 31], the effect of PTH inhibition of CXCL12 secretion by MC3T3-E1 cells (preosteoblasts) was confirmed. In addition, the mRNA expression of *CXCR4* and *PTHrP* was markedly reduced in primary tumors dissected from mice treated with PTH. Collectively, these experiments clearly demonstrated that treatment of osteoblasts with intermittent PTH reduced migration of both human and murine BrCa cells and altered the expression of several genes implicated in metastases, thereby rendering the bone marrow hematopoietic niche less favorable for the homing and colonization of cancer cells.

Patients with osteolytic bone metastases currently are not treated with intermittent PTH because concern has been associated with the use of this drug due to the development of osteosarcoma in preclinical studies [63]. However, the Osteosarcoma Surveillance Study, an over 10-year surveillance study initiated in 2003, is a postmarketing commitment to evaluate a potential relationship between teriparatide and development of osteosarcoma and has not detected a pattern indicative of a causal association between teriparatide treatment and osteosarcoma in humans [64, 65]. Nonetheless, the antitumor effects of PTH provide proof of principle for the use of bone-anabolic agents against MM or BrCa osteolytic bone disease. These findings warrant further investigation for the safety and efficacy of teriparatide or abaloparatide in MM or BrCa patients.

### 5.2. Allosteric Modulators of CaSR

Ligands that activate the CaSR termed as calcimimetics include agonists (type I) and positive allosteric modulators (type II) (Table 2). The action of calcimimetics is to inhibit the secretion of PTH. CaSR antagonists are calcilytics that act as negative allosteric modulators and stimulate the secretion of PTH [66]. Although both positive and negative allosteric modulators of the CaSR are already in development, currently, only the positive allosteric modulators are approved for use in humans. Cinacalcet was the first US FDA-approved allosteric GPCR modulator in 2004 and is used for the reduction of hypercalcemia in patients with parathyroid carcinoma and severe primary hyperparathyroidism, who are unable to undergo parathyroidectomy. The treatment of secondary hyperparathyroidism in patients with end-stage renal disease on maintenance dialysis therapy by cinacalcet was also approved [67]. In 2017, etelcalcetide (AMG 416), a second-generation calcimimetic agent, was approved by the US FDA for the treatment of secondary hyperparathyroidism in patients with chronic kidney disease on hemodialysis. It is established that excessive secretion of PTHrP by tumors stimulates osteoclastic bone resorption and promotes renal proximal tubular reabsorption of calcium, leading to hypercalcemia of malignancy [68, 69]. The applications of cinacalcet occurred subsequently in patients with bone metastases of renal cell carcinoma [70] and BrCa [71] that cause hypercalcemia. Asonitis and colleagues recently reported that a patient with metastatic BrCa developed severe hypercalcemia in the disease progression [71]. Medical treatment with bisphosphonate (zeledronate) and RANKL inhibitor (denosumab) failed to lower the elevated serum calcium level. Cinacalcet was then added to the medication and effectively reduced tumor-mediated hypercalcemia and maintained the calcium levels within the normal range in this patient.

The mechanisms of cinacalcet effect for use of treatment of BrCa patients with hypercalcemia are not completely understood. CaSR is expressed not only in the parathyroid glands and kidneys but also in bone cells and metastatic BrCa cells. Expression of CaSR promotes PTHrP secretion in human BrCa cells [33] and increases osteolytic bone metastases associated with decreased bone formation and increased tumor burden in the mouse intratibial model [72]. *In vitro*, activation of the CaSR with Ca^2+^ or positive allosteric modulator increased PTHrP secretion by BrCa cells [26, 33]. Frees et al. reported that CaSR antagonist NPS 2143 was able to reverse Ca^2+^-induced increase in cell adhesion, migration and proliferation in renal carcinoma cells transfected with the CaSR plasmid [73]. However, the effects of CaSR antagonist on CaSR-mediated BrCa bone metastases and osteolytic bone lesions have not been reported yet. The findings from Colloton and colleagues may help understand the pharmacologic effect of cinacalcet on the decrease of hypercalcemia in patients [74]. The mechanism by which cinacalcet lowered serum calcium was investigated in parathyroidectomized rats by injection of high-dose PTHrP to generate hypercalcemia [74]. Cinacalcet attenuated PTHrP-mediated elevations of ionized calcium in the blood, which were accompanied by increased plasma calcitonin. Cinacalcet was also found to attenuate PTHrP-mediated increase of serum calcium in mice bearing C26-DCT colon tumors [74], which do not express CaSR. These results suggest that the cinacalcet-mediated decrease in blood calcium is not the result of a direct effect on tumor cells but rather is the result of increased calcitonin release.

Several calcilytic compounds that are antagonists of CaSR have been evaluated as orally active anabolic therapies for postmenopausal osteoporosis, but clinical development of all of them has been abandoned because they lacked tissue selectivity and clinical efficacy [67]. However, the administration of calcilytics for inhibition of CaSR activation has recently been aroused as promising therapies in other diseases such as chronic obstructive pulmonary disease or allergic asthma [75, 76] and could also be used for preventing osteolytic bone metastases.

## 6. Conclusion

BrCa bone metastases are common in advanced malignancy. Despite the developments in both anticancer and bone-targeted therapies in recent years, new therapeutic strategies remain to be considered. Both PTH1R and CaSR participate in the bone-tumor vicious cycle and influence the skeletal metastatic niche. Teriparatide and abaloparatide have been successfully applied in osteoporosis. CaSR agonist cinacalcet was effectively used to lower the blood calcium level in BrCa patients with hypercalcemia. The second-generation CaSR agonist etelcalcetide (AMG 416) was recently approved for the treatment of secondary hyperparathyroidism. ^223^Radium dichloride is a calcimimetic that binds preferentially to a newly formed bone in areas of bone metastases, is the first alpha-emitting radionuclide to be developed for clinical use, and is approved for treatment of castration-resistant prostate cancer and symptomatic bone metastases [77]. Those PTH1R- or CasR-based agents influence PTHrP-PTH1R and Ca^2+^-CaSR signaling pathways in the vicious cycle and could be used for preventing bone metastases and their associated bone destruction although their safety and efficacy need to be further evaluated.

## Figures and Tables

**Table 1:** Similarity and difference between PTH and PTHrP.

	PTH	PTHrP
Production	Parathyroid glands	All cells especially tumor cells including breast cancer cells
Protein size	84 amino acids	PTHrP is comprised of 139, 141, or 173 amino acids
Action mechanism	Acts as an endocrine factor	Acts as an endocrine, paracrine, autocrine, or intracrine factor
Binding to receptor	Binding to both PTH1R and PTH2R	Only binding to PTH1R
Nuclear localization sequence	No	Yes, increase of tumor proliferation
Promotion of bone resorption	Yes	Yes
Increase of renal tubular reabsorption of calcium	Yes	Yes
1-*α*-Hydroxylase	Activates its activity to form 1,25 dihydroxy-vitamin D and then promotes calcium absorption in the intestine	No
Hyperparathyroidism	Primary and secondary hyperparathyroidism	No
Humoral hypercalcemia of malignancy	No, except parathyroid carcinoma	Yes

**Table 2:** Comparison of PTH1R and CaSR.

	PTH1R	CaSR
GPCR	Class B family	Class C family
Receptor size	Human PTH1R has 593 amino acids	Human CaSR has 1078 amino acids
Expression	Mostly in osteoblast and kidney, also in cartilage, normal breast epithelial cells, and some breast cancer cell lines	Parathyroid glands, kidney, bone, normal breast epithelial cells, and BrCa cells
G protein	G*α*s, G*α*q, G*α*i, and G_12/13_	G*α*i, G*α*q, G*α*s, and G_12/13_
Agonist	PTH and PTHrP	Type I: inorganic or organic polycations (Ca^2+^ and Gd^3+^), polyamines (spermine and spermidine), and aminoglycoside antibiotics (neomycin) Type II: positive allosteric modulators (calcimimetics)—cinacalcet, NPS R-467, NPS R-568, and AMG 416
Antagonist	PTH(7–34)	Negative allosteric modulators (calcilytics): NPS 2143
Application in treating BrCa bone metastases	Intermittent PTH(1–34) prevents BrCa bone metastases in mouse models	Cinacalcet is able to treat severe hypercalcemia caused by BrCa bone metastases
